# THE RESOURCE EXCHANGE NETWORK: A MODEL FOR TRANSFORMATIVE CHANGE IN GERIATRIC CARE

**DOI:** 10.1093/geroni/igz038.2976

**Published:** 2019-11-08

**Authors:** Phillip G Clark, Phillip Clark, Christine Ferrone, Faith Helm, Alexandra Morelli

**Affiliations:** University of Rhode Island, Kingston, Rhode Island, United States

## Abstract

Transformational efforts to redesign the care system for older adults call for the development of novel partnership models incorporating academic institutions, primary care networks, and community-based organizations. The Rhode Island Geriatric Workforce Enhancement Program (RI-GWEP) has utilized the “resource exchange model” to develop innovative, interdisciplinary, and integrated projects combining educational resources, clinical expertise, programmatic experience, and impact evaluation. With particular emphasis on the challenges of developing interprofessional education spanning the traditional gaps between disciplines and departments, this presentation emphasizes the critical conditions, components, and capacities of such a collaborative network. These include: (1) mission dominance, (2) barter exchange, (3) partnership investment, and (4) interpersonal relationships. RI-GWEP’s experience provides insights into the conceptual foundation of geriatrics networks and specific, concrete examples of projects incorporating these principles. Implications for the development of networks in other settings include consideration of particular: (1) projects, (2) people, (3) places, (4) personalities, and (5) possibilities.

